# Gleanings in Science and Medicine

**Published:** 1893-08-05

**Authors:** 


### Gleanings in Science and Medicine.

A somewhat curious dinner has just taken place at Grenoble, in Dauphiné, France. This was a dinner to which were bidden the fattest men in Europe, the list of names was drawn up, and invitations issued accordingly. The spectacle thus presented on the night fixed was said to be entirely unique, and the gathering of the portly was described as of so successful a character that the festival will become an annual one. The admittance to the dinner was restricted to such persons as could boast of a weight not under 220 pounds.

Paris is exhibiting a new utilisation of glass for household purposes, which takes the form of window and bed curtains. This invention is said to be possessed of many virtues—as a window curtain it is pretty and effective, and does not obscure the light, whilst as a bed curtain it ensures a certain immunity from the danger of a conflagration of such persons as indulge in the pernicious habit of reading in bed. The glass for these new hangings is manufactured in small coloured squares, each in zinc frames, having tiny hooks at the sides by which they are hung to one another.

The picturesque is not always the sanitary, and many an historical residence bestowes its benefits more on the sight-seer rather than on those who dwell therein. Travellers flock in numbers from the new world to admire and envy our beautiful buildings rendered poetic by decay. What appeals more vividly to the imagination than a moat? A moated grange is generally the wings upon which the budding romancer takes his first flight into literature. Admiration receives a little shock, however, when we are told that the pretty pool into which we are gazing, studded with water lilies and gay with water fowl, is in reality the cesspool of the interesting castle standing above. This is a very real and unpleasant fact, and our wonder is further excited as to the why and wherefore the inhabitants are in health, and typhoid does not seize his victims. The laws of nature may be ignored for a considerable time, but it seldom happens that unfortunate victims do not eventually pay for such negligence.

In the pages of a weekly contemporary we find the question raised, whether germs, in time, become modified like higher organisms. Up to the present time, speculations on the past and the future of bacilli must necessarily be of a somewhat hazy charater, but quite recently there has been made a steady advance in the investigation of certain germs, in particular the cholera form. One experimenter on this subject, in comparing these germs together, some coming from Paris, and some from Hamburg, found an exact similiarity in their structure, but at the same time they were found to differ widely from the cholera bacilli common in India. The difference being in all probability due to the climatic influence on the European and the Indian. This theory rather points to the fact that this particular germ, in becoming attenuated through circumstances, may have lost some of its disease-producing power, a theory which if carried furthur, places the germ in the category of things extinct, amongst diseases which, like the Black Death and the Plague, enact now no longer a devastating work on human beings. This is a hopeful theory, but the writer of the article setting it forth does go so far as to admit his speculation to be a bold one—and he barely asks more for it than that it be regarded in the light of a suggestion.

It is hard to prevent people poisoning one another, if such be their wish, but a case which occurred not long ago certainly suggested that there are means yet unemployed which if brought into action might, anyhow, put some small obstacles in the way of the thus evilly-intentioned persons. Pessimists are somewhat too fond of the philosophy which relates to spilt milk. If none cared at all for adverse fortune there would be more of it, not less, and the law of prevention would not be brought into play at all. The case to which reference was made above, was that of a man who died under circumstances which led suspicion to be directed against those who surrounded him at the last. His regular servants having been dismissed by doctor and nurse, who were the sole persons to witness the death-bed scene, as also the illness preceding it, the case was one which fairly warranted police interference—certain unaccountable facts having come to light concerning it. It was suggested at the time that for the safety of the public, a medical officer, paid from general funds, should be provided in every district, whose duty it should be to inspect any such corpses as are not submitted to a post-mortem examination. Were it thus made compulsory by law that no corpse could be disposed of without this independent medical testimony, such cases as we refer to would find it hard to repeat themselves.

If we were asked which of the winds that blow is the most frequent visitant of our shores, it is doubtful if we should have an answer ready, because to most of us it is not easy to tell in what direction the wind blows unless we have the special aid of a weather-cock to guide us, or unless we feel certain physical effects upon our system. Some people (keen observers these in the vast book of Nature) go so far as to say that a glance from the window, independently of any sensation, tells them through the relative clearness of the light, by the atmospheric appearance of things around, in what direction the wind is. The laws which guide these observations are not easy of interpretation through words, but certain it is that sharp clean cut shadows belong to an E. wind day, changeful lights and shadows to the S. and W. winds, and a bleak, monotonous, undefined labouring to the N. wind. But, as we say, these are only relative terms, and their reading is confined to the few. As a matter of fact, the most constant wind blowing over England is the South-west. The Register kept by the Royal Society, taking an average of ten years, from London observations, has attested to this fact. The order of the prospective frequency of the winds as given by this Register we append below:—N.E., 58 days; N.W., 50; W., 43; S.E., 32; E., 26 S., 18; N., 16; whilst the South-west dominated by 112 days in the yearly cycle.

